# Comparative Gastric Morphometry of Muong Indigenous and Vietnamese Wild Pigs

**DOI:** 10.1100/2012/894952

**Published:** 2012-10-11

**Authors:** Pham Hong Trang, Peck Toung Ooi, Abu Bakar Zakaria Zuki, Mustapha Mohamed Noordin

**Affiliations:** ^1^Faculty of Veterinary Medicine, Universiti Putra Malaysia, Selangor, 43400 Serdang, Malaysia; ^2^Faculty of Veterinary Medicine, Hanoi University of Agriculture, Gia-Lam District, Hanoi 010000, Vietnam

## Abstract

It is hypothesized that despite sharing a similar habitat, the Muong indigenous and Vietnamese wild pigs may reveal different gastric morphology. Due to the protective nature of procuring these pigs, a total of 12 Muong indigenous pigs and nine Vietnamese wild pigs stomach collected post mortem were analysed for selected biometric parameters and histology. The result indicated that the stomach of the Vietnamese wild pig is broader with a bigger capacity and greater proportion of proper gastric glands. Interestingly, the stomach mass correlated well with live body weight in both breeds apart from possessing similar histomorphometry of the gastric gland regions. On the other hand, the thicker (*P* < 0.05) submucosa in the Vietnamese wild pig is attributed to the presence of numerous loose connective tissues, abundant blood vessels, adipose tissues and nerve plexus. The appearance of lymphoid follicles underneath the tubular gastric glands in the Vietnamese wild pig exceeded that of Muong indigenous pigs. This finding suggested that the difference in feeding behavior as well as immunity. In conclusion, adaptations found in the Vietnamese wild pig indicated that this breed is equipped with a bigger and effectively functional stomach to suit its digestive physiology and immunity in the wild.

## 1. Introduction

The demand for “exotic” pork in Vietnam is continuously on the rise since disappearance of primary forest has led to a decreased wild boar population [1]. Furthermore, the intensively reared Moung indigenous and Vietnamese wild pigs yielded frustrating results to meet the local demand. This is with respect to various production intervention used were more of by trial and error in trying to increase their productivity without proper understanding of their actual anatomy and possibly physiology that has hampered the effort. It is believed that lack of data on gastrointestinal morphometry of these indigenous and wild pigs [2] is partially responsible for the failure improving the productivity of these pigs to an acceptable economic scale [3, 4]. This is especially so since gastric biometry is known to affect the efficiency of feed conversion and thus growth rate and survival [5–7]. The need for growth and survival has transformed the porcine stomach [8] to have a higher proportion of functional gland where the cardiac gland region covers about 50% of the total mucosal surface [9].

It is believed that the attempt of this study is to unveil the gastric histometry of Vietnamese indigenous, and Vietnamese wild pigs would enhance a better understanding towards rearing these highly demanded pigs on a commercial scale since the morphology of the gastrointestinal system does reflect the actual demand for optimum growth and thus productivity of these pigs.

## 2. Materials and Methods

### 2.1. Animals

The indigenous and wild pigs used in this study were the Muong indigenous and Vietnamese wild pig, respectively. Owing the strict conservation regulatory measures, the Wildlife Authority of Vietnam (WVA) has only permitted the usage of a total of nine Vietnamese wild pigs in this study. These wild Vietnamese pigs used were those trapped by WVA while invading the plantation during harvesting season. All Muong indigenous pigs were purchased from a farmer in the Hoa Binh province (Luong Son district), Vietnam.

 The live body weight (kg) was recorded using a commercial scale with a 0.1 kg graduation. The body length (cm) was made using measuring tape laid flat on a straight line on the back of animal from the snout to the base of tail. The gastrointestinal tract studied was comprised of the mass and length of the stomach, small intestines, and large intestines, however in this paper only the data on the stomach is presented.

### 2.2. Gross Gastric Morphometry

In this study, the stomach was designated as the region from the lower esophageal sphincter to the pyloric sphincter and its weight (g) was taken without the ingesta. Stomach was taken immediately after the pigs were sacrificed by overdose of barbiturate which was later separated free from esophagus and small intestine with a knife. The designated stomach segment was milked and massaged gently to expel the ingesta and then let to drip via gravity. The length of major and minor curvature of stomach was taken using a measuring tape commencing from lower esophageal sphincter to pyloric sphincter without any undue stretching and the total mass was weighed on a balance.

### 2.3. Gastric Histomorphometry

After the measurement of the superficial dimension, the inner surface of stomach was revealed by opening through the lesser curvature. All remaining contents were gently removed under slow running tap water. The distribution of the regions of the stomach was later estimated prior to histology sampling. Samples were selected (3 × 4 cm) from cardiac, gastric, and pyloric gland regions on greater curvature and fixed in 10% buffered formalin for further histomorphometry studies.

Tissue selected from afixed area was trimmed (≤3 mm thick) and fixed again in fresh 10% buffered formalin for 48 hours before being processed in the routine manner.

All slides were stained with periodic acid Schiff (PAS), Alcian blue pH2.5 (AB), and hematoxylin and eosin (H&E). Histomorphometry assessment was made on 20 fields of each slide via a battlement manner at the X5 magnification under a light microscope (Leica, Japan) for the muscularis externa, submucosa, and mucosa.

### 2.4. Statistical Analysis

All data were expressed as mean **± **standard error (SE) and subjected to selected tests using SPSS (ver. 19) where only *P* < 0.05 is considered significant. The comparative histometry of the stomach was obtained by using independent *t*-test samples. Linear regression correlation was employed to assess the relationship between the weight of stomach and its dimensions.

## 3. Results

### 3.1. Gross Gastric Morphometry

**Figure 1:**
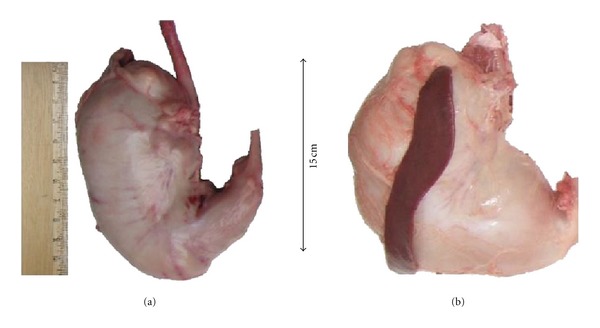
Photograph of the intact stomach at post mortem of the (a) Muong indigenous and (b) The Vietnamese wild pigs. Note the mark differences in terms of shape where it is much more circumscribed in (b). In general, the shape of the stomach of the Muong pig closely resembles that of the domestic pig.

Figure 1 shows the gross morphology of the stomach where the shape of the Vietnamese wild pig was much more circumscribed. Furthermore, its minor curvature was much longer and obtusely-angled than that of the Muong indigenous pig. However, the pyloric part of the Muong indigenous pig was slender and its terminal portion was closer to the esophageal sphincter and resembled that of the domestic pig.

**Figure 2:**
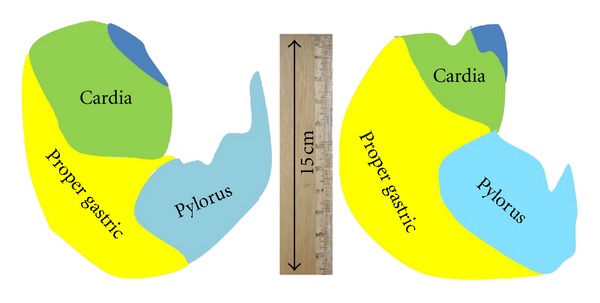
The comparative distribution of gastric regions between both breeds.

As tabulated in Table 1, the greater curvature of stomach of the two breeds showed no significant differences (*P* ≥ 0.05) with only being 3% longer in Vietnamese wild pigs. On the other hand, the lesser curvature of stomach yielded a remarkably (*P* <  0.05) shorter measurement by nearly 20% in the Muong indigenous pig. The distribution of the respective regions is shown in Figure 2 where the Vietnamese wild pig has a larger proper gastric gland. 

**Figure 3:**
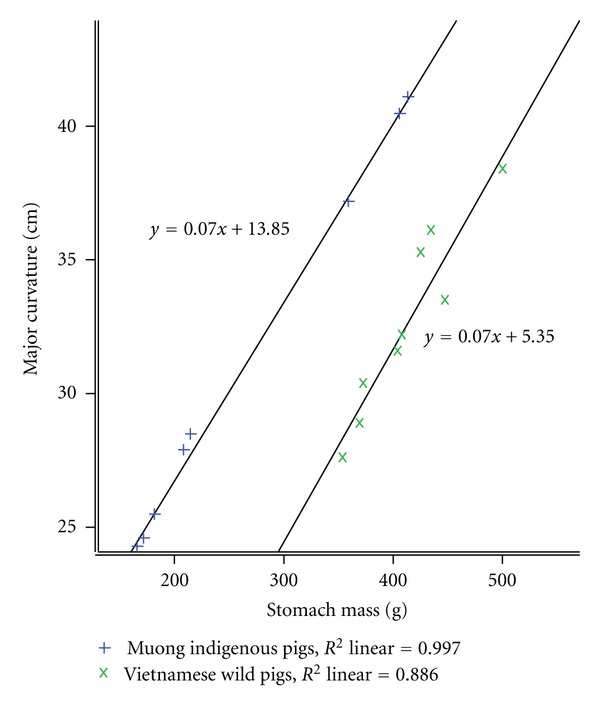
Comparative correlation of major curvature to stomach mass.

**Figure 4:**
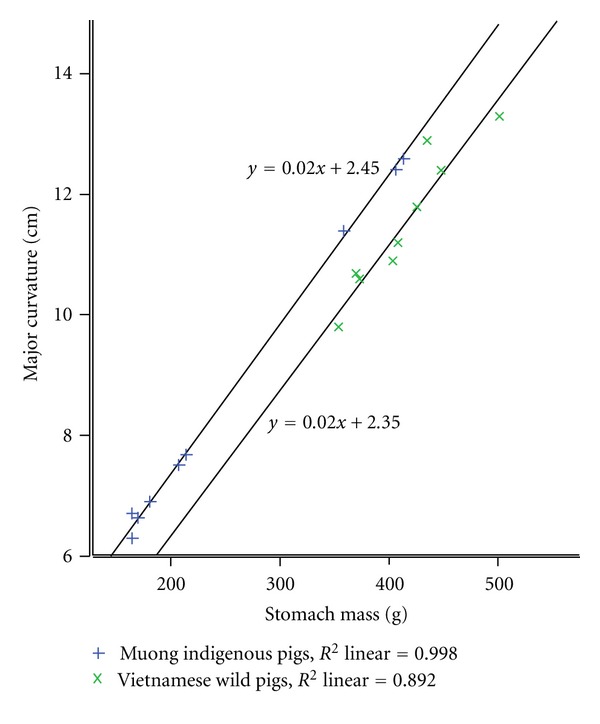
Comparative correlation of minor curvature to stomach mass.

In order to manifest the relationship of stomach's dimensions to its weight, linear regression lines have been expressed as shown in Figures 3 and 4. The positive tendency of regression lines demonstrated that the dimensions of major and minor curvature highly corresponded to its stomach mass (*R* ^2^ linear ≥0.88). Comparatively, both breeds showed significant (*P* < 0.05) difference with regards to this relationship.

### 3.2. Gastric Histomorphometry

The microscopic structure of stomach in both breeds is clearly delineated into three different glandular regions and consisted of three main layers, namely, the external muscularis, submucosa, and innermost mucosa. The histomorphometry of functional regions of the cardiac, proper gastric, and pyloric glands are shown in Table 2. In both breeds, the thickest and thinnest layer was the muscularis and mucosa, respectively. 

**Figure 5:**
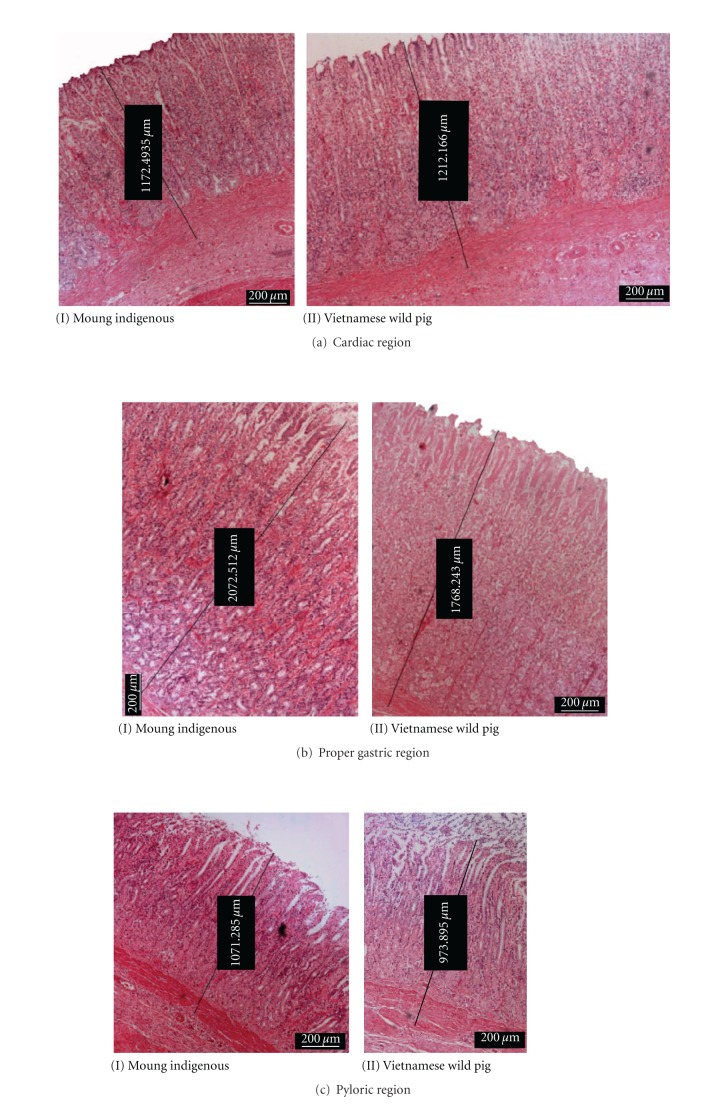
photomicrograph of H&E staining showing tissue differences in thickness between both breads.

**Figure 6:**
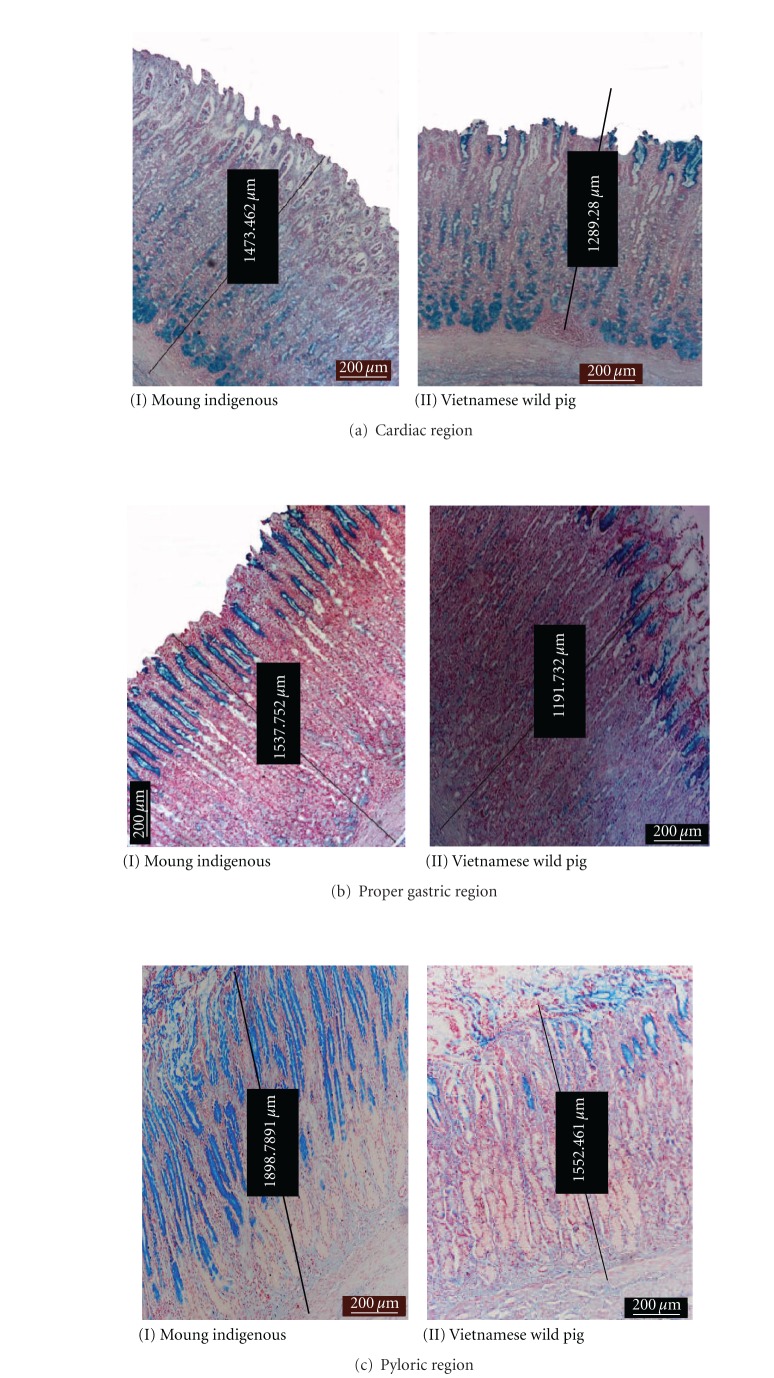
Photomicrograph of AB staining showing different affinity of positive areas at various sites.

**Figure 7:**
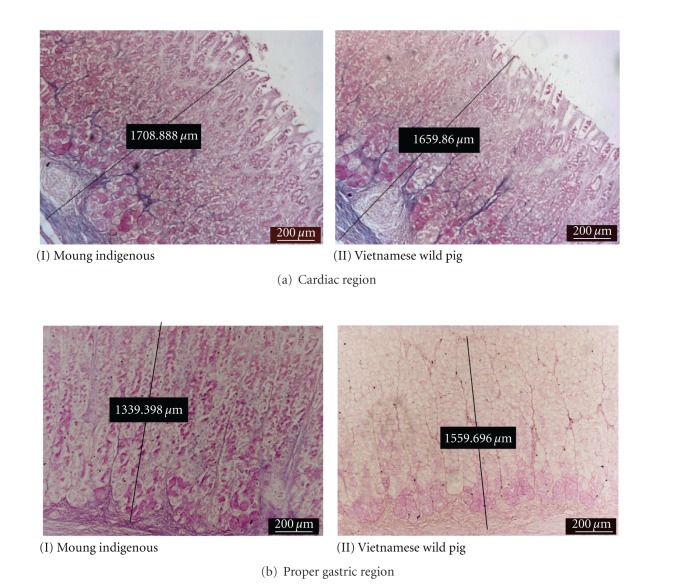
Photomicrograph of PAS staining showing different affinity of cells towards the stain.

Figures 5, 6, and 7 show the photomicrographs of the respective regions of the stomach in both breeds stained by H&E, AB, and PAS, respectively. Microscopically, the three layers of the muscularis were clearly discernible where the outermost was the thin longitudinal smooth muscle and occupied nearly a third of the total area (Figure 5). Covering about 2/3 of the total thickness of the muscularis was the middle circular muscle. The innermost layer was the oblique muscular that adjoins to the submucosa. This layer was thin and found to be not well-differentiated from circular layer in some sections. In addition, the myenteric plexus and blood vessels are sometime located between the two outer muscularis layers. 

The submucosa layer presented about 10% of the total thickness of the gastric and was composed of dense irregular connective tissues intermingling with numerous large blood vessels, white adipose tissues, and lymphatics.

Tunica mucosa was thickest in the proper gastric (Figure 5) and thinnest in cardiac gland region (Figure 5). In general, the bulk of the stomach in both breeds is predominated by the proper gastric gland followed by the pyloric and later the cardiac glands.

#### 3.2.1. Cardiac Glands

The mucosa of this region was significantly thicker (*P* < 0.05) in Muong indigenous pigs compared to the Vietnamese wild pig. The volume of gastric pits was nearly half and showed numerous deep empty furrows. The tubular gland body was relatively short, occupied a lower quarter of tunica mucosa and stained moderate-to-strong reaction with AB (Figure 6) but deeply with PAS (Figure 7). While the small amount of epithelial cells on surface reacted positively with AB and PAS, the neck area was almost unstained. There were the lymphoid tissues beneath the coiled glands.

As depicted in Figure 6, the lymphoid follicles were well developed and even reached a dimension of more than 500 *μ*m in diameter. In this region, the infiltration of lymphoid tissue observed in Vietnamese wild pigs was much stronger and more frequently seen than in the Muong counterpart (78% versus 17%).

#### 3.2.2. Gastric Glands Region

In this region, the thickness of the submucosa in the Muong indigenous pigs was greater (*P* < 0.05) than that of the Vietnamese wild pig (Figure 5). The proper gastric glands are tubular branches and longest among stomach's glandular regions, predominated 80% of the mucosal density. The gastric pits were the shallowest in comparison to other areas, and this is the only place where the pit was not be able to spread or branched and only connected to the neck of gastric gland at the proximal one-fifth. 

The base of the gland stained moderately with PAS and weakly with AB (Figures 6 and 7). Apart from connective tissue, the chief regions showed no reaction to both AB and PAS stain, while the neck of tubular gland was AB positive. Almost all nuclei of cells were deeply stained red by nuclear fast red counterstain (Figure 6). Meanwhile, the majority of epithelial cells covering gastric pits positively reacted with AB revealing the presence of different glycoproteins.

#### 3.2.3. Pyloric Glands Region

In Muong indigenous pigs, the mucosa of this region was insignificantly thicker than that of Vietnamese wild pigs (Figure 5). The gland was short, tubular, branched, and with a density of less than 50%. The empty gastric pits was long, branched, grooved, and sometime opened to gastric gland. The muscular laminae were less dense and covering group of pyloric glands.

## 4. Discussion

Grossly, the main feature that distinguishes the Vietnamese wild pig stomach from those of the Muong and other Suidae is its shape being more globose and rounded. It is believed that the globose-shaped stomach would enhance greater capacity for storage with respect to its survival in the wild. Under this condition, not only there is scarcity but also the likelihood of getting enough especially with the intense deforestation and destruction of its habitat [1].

Stomach is well known to be the temporary storing place and first step in breaking up of raw ingested feed material. Its dimensions and weight varies depending on breeds, age, body size, and the availability of the nourishment [10, 11]. The greater the percentage of the wild genotype is, the higher the ratio of stomach to body weight is [6] as seen in the Vietnamese wild pig as reported here. For example, this rate was ranged from 0.57 to 0.68% in commercial pig breed [12], reached 0.74% in Lithuanian indigenous pigs [6], and approached 1.94% in Collared peccary [7]. However, the increase of Vietnamese wild pig genotype in crossbred effected on relative empty stomach weight but the difference was not significant between *½* and ¼ wild boar after receiving the same standard concentrate feed [6].

Considering the influence of pure wild gene, we found that Vietnamese wild pigs have a higher ratio of stomach/body compared to Muong indigenous pigs. Analysis of the stomach ingesta of the Vietnamese wild pig [13–15] proved that their diet could not meet the energy requirements. Therefore, as found in this study, the Vietnamese wild pigs have to consume greater amount of food and thus require a larger stomach volume.

The result of stomach dimensions once again demonstrated that the change of physiology of stomach is the consequence of the impermanent in “quantity and quality” of food source [16] and the difference in the location of regression illuminates the dissimilarity degree of adaptation [17].

 In general, the *Sus scrofa *cardiac, gastric, and pyloric glands unequivocally occupy approximately 33% of the total glandular stomach area [18]. However, in babirusa almost 70% of the total surface area of the glandular stomach is occupied by the cardiac glands [19]. Currently, reviews on the gastric anatomy of Vietnamese wild pigs indicated that they were very similar in their anatomy to that of the domestic pig [20]. The findings in this study as opposed to the documented reports [17–20] showed that at least the gastric morphometry of the Vietnamese wildpigs in Vietnam have not probably undergone any significant change.

In this study except for the pyloric gland which is similar to other pigs, the bulk of the glandular stomach is occupied by the gastric glands in both breeds. However, the Vietnamese wild pig tends to even differ further by having the least area of cardiac gland. Thus, this is the first study to indicate that the gastric morphometry of the Vietnamese wild pig is markedly different from other porcine breeds. It has been shown that the differences in the morphology of the GIT possibly explained the variation in the digestive transit time, gastrointestinal microbial activity, nutrient, digestibility, and in the overall efficiency of energy use [21]. Findings in the Vietnamese wild pig could explain the need of having a much more digestively effective and voluminous stomach to ensure greater chances of survival in the wild. 

Both studied subjects showed the identical morphology of functional gland regions with most of mammalian breeds described elsewhere. The volume of muscularis externa that fluctuated between 0.44 and 0.5 is remarkably higher than in other omnivores (0.180) [17] and much broader than true carnivores (0.07) [22]. This difference is believed to be affected by actual body size and feeding behavior [23]. Additionally, the significant increase of tunica muscularis imply the necessity of increasing surface region for the accurate arrangement of the flexible muscle tissue creating spaces for soft inner parts [7, 17]. Evidently, the metabolic activities require the effectiveness of the chemical and the mechanical process, therefore the smooth muscle fibers are needed for successfully adaptation [17].

The staple diet of the Muong indigenous pigs is composed of plant materials such as potato buds, taro leaves, banana trunks, and the indispensable component, the rice bran [3]. Conversely, although the dietary composition of Vietnamese wild pigs still remains to be investigated, it is believed that deforestation [1] might have to begin altering its previous diet and thus gastric morphometry. The thicker submucosa layer of proper gastric gland areas in Muong indigenous pigs could have arisen from the abundant blood vessels, loose connective tissues, and white adipose tissues. However, it is difficult to ascertain if this reflects the vigorous metabolic activities adapting to the different quality of food-supply [24]. Thus, further studies on digestive physiology should be conducted in both breeds to correlate the gastric anatomy and its function. 

Surprisingly, the histological features of the functional gland regions in both breeds were similar to those of domestic pigs and most mammals [18, 23, 25, 26]. However, differences existed in terms of percentage distribution of different regions so as to improve digestion based on diet (Figure 2). 

The cardiac glands are considered to be the main site for mucus producing accompanied with the epithelial columnar cells in the surface of gastric mucosa [18, 27]. The strong AB positive reaction at the base of this region in both breeds differed from babirusa [19] and is similar to that of camel [25]. This indicates that the area is composed of mainly acid mucins [25] as opposed to that of Leus et al. [19] to be of neutral mucins. Furthermore, the cardiac gland region in babirusa's stomach covers more than 70% of mucosal surface area as opposed to that of Muong indigenous (29%) and Vietnamese wild pigs [17]. Similarly, in both studied breeds, the bulk (more than 40%) of the stomach is composed of the proper gastric glands. 

Nordman et al. [28] proved that the fundamental glycoprotein of mucin produced from epithelium and gland region was dissimilar and differ among glands themselves. The main feature making these differences includes buoyant density, apoprotein structure, and carbohydrate substitution. Furthermore, the consequence is uneven staining in tissue sections as also seen in this study.

An unexpected finding in this study was the frequent appearance of lymph nodes in lamina propria of gastric gland region. The presence of lymphoid tissues in gastrointestinal tract such as Peyer's patch in small intestine can be considered as normal histology structure fixed genetically [16]. The study on gnotobiotic pig revealed normal structure of lymph nodes in submucosa of cardiac region of stomach and could migrate to mucosa layer [29]. The similar result can be found in monkey [30]. Therefore it is probable that the stomach structure was not only designed for digestion but also genetically immunology contributed. 

On the other hand, Owen and Ermak [31] stated that the mucus barrier created physically in stomach is to keep away harmful microorganisms, therefore the number of lymphoid tissues in healthy stomach is rare. Moreover, in rats and dogs the infiltration of lymphoid tissues underneath lamina propria increased in number and size throughout the infection period [32, 33]. Additionally, Owen and Ermak [31] pointed that although the adaptation of different sites could be modified but all epithelial accord mucosa are facilitated for antigen uptake. This is especially so in the Vietnamese wild pigs which were never vaccinated or are continuously exposed to pathogens and require their own immune system to combat the infection.

Therefore, investigations on dietary as well as the definition of the adaptabilities of Vietnamese indigenous and Vietnamese wild pig would explain the factors affecting their growth performance. This will pave the way towards exploiting the required factors in transforming these breeds into a much more economically commercialized pig.

## 5. Conclusion

Although, the Muong and Vietnamese wild pigs do differ in several histological aspects of the stomach, that of the latter greatly differs from that of other pigs. It is greatly voluminous and is composed mainly of the gastric glands. Such adaptation is believed to render a greater chance of survival due to the uncertainty of procuring a constant supply of food and that this pig is a forestomach fermenter. Likewise, the presence of more and larger lymphoid follicles also signifies it much more vigilant immune system which remains to be further investigated. However, although still preliminary, the findings are believed to have paved the way in producing these pigs at a commercial scale.

## Figures and Tables

**Table 1:** Comparative major and minor curvature of stomach (Mean ± SE).

	*n*	Major curvature (cm)	Minor curvature (cm)
Muong indigenous pigs	12	31.7 ± 1.88	9.1 ± 0.70^a^
Vietnamese wild pigs	9	32.7 ± 1.17	11.5 ± 0.38^b^

^a,  b^Values within columns bearing different superscripts differ at *P* < 0.05.

**Table 2:** Comparative histomorphometry of stomach gland regions (*μ*m; Mean ± SEM).

Region	Muong indigenous pigs *n* = 12	Vietnamese wild pigs *n* = 9
Cardiac glands		
Tunica muscularis	1156.4 ± 78.95	1106.5 ± 84.37
Submucosa	174.4 ± 19.52	158.7 ± 62.75
Tunica mucosa	947.2 ± 32.27^a^	912.5 ± 63.79^b^

% of total stomach surface*	28.7 ± 0.99^a^	16.7 ± 0.56^b^

Proper gastric glands		
Tunica muscularis	1412.1 ± 97.67	1369.2 ± 90.3
Submucosa	292.4 ± 28.41^a^	186.4 ± 14.51^b^
Tunica mucosa	1488.9 ± 100.31	1459.7 ± 45.39

% of total stomach surface*	40.1 ± 1.34^a^	46.5 ± 1.56^b^

Pyloric glands		
Tunica muscularis	1177.6 ± 81.36	1163.1 ± 41.36
Submucosa	163.9 ± 17.84	126.2 ± 21.07
Tunica mucosa	1134.5 ± 39.42	1096.5 ± 75.92

% of total stomach surface*	31.1 ± 1.20	36.8 ± 1.22

^a,  b^Values within row bearing different superscripts differ at *P* < 0.05.

*These data were used to construct Figure 2.
